# The Cytoplasmic Actins in the Regulation of Endothelial Cell Function

**DOI:** 10.3390/ijms22157836

**Published:** 2021-07-22

**Authors:** Vera B. Dugina, Galina S. Shagieva, Anton S. Shakhov, Irina B. Alieva

**Affiliations:** 1 A.N. Belozersky Institute of Physical and Chemical Biology, Lomonosov Moscow State University, 119992 Moscow, Russia; vdugina@iname.com (V.B.D.); galya_shagieva@yahoo.com (G.S.S.); sh.anton90@yandex.ru (A.S.S.); 2 Federal Research and Clinical Center of Physical-Chemical Medicine of Federal Medical Biological Agency, 1a Malaya Pirogovskaya St., 119435 Moscow, Russia

**Keywords:** endothelial cell, endothelial barrier function, cytoskeleton, non-muscle actin isoforms, β-actin, γ-actin

## Abstract

The primary function of the endothelial cells (EC) lining the inner surface of all vessels is to regulate permeability of vascular walls and to control exchange between circulating blood and tissue fluids of organs. The EC actin cytoskeleton plays a crucial role in maintaining endothelial barrier function. Actin cytoskeleton reorganization result in EC contraction and provides a structural basis for the increase in vascular permeability, which is typical for many diseases. Actin cytoskeleton in non-muscle cells presented two actin isoforms: non-muscle β-cytoplasmic and γ-cytoplasmic actins (β-actins and γ-actins), which are encoded by ACTB and ACTG1 genes, respectively. They are ubiquitously expressed in the different cells in vivo and in vitro and the β/γ-actin ratio depends on the cell type. Both cytoplasmic actins are essential for cell survival, but they perform various functions in the interphase and cell division and play different roles in neoplastic transformation. In this review, we briefly summarize the research results of recent years and consider the features of the cytoplasmic actins: The spatial organization in close connection with their functional activity in different cell types by focusing on endothelial cells.

## 1. Introduction

The vascular endothelium is formed by the monolayer of specialized tightly adjoining and connecting to each other endothelial cells (EC). EC form a selective semi-permeable barrier between the interior space of blood vessels and the underlying tissues. The endothelial barrier regulates liquid and macromolecule transport between the blood and the interstitial space and is highly susceptible to regulation by various stimuli of physiological and pathological origins. EC barrier integrity is critically dependent upon intact cytoskeletal structure and cell junctions. Reorganization of the endothelial cytoskeleton, especially and the actin system, results in alteration in cell shape and its compression and provides a structural basis for an increase in vascular permeability, which has been implicated in the pathogenesis of many diseases including asthma, sepsis, acute lung injury ischemia, and diabetes.

Actin is one of the most abundant proteins in eukaryotic cells. The ability of actin to polymerize and interact with enormous number of other proteins enables it to perform many different functions. All the cytoskeletal proteins are capable of polymerization, however, the exchange between monomeric and polymerized actin in the cytoplasm occurs rapidly and the exchange time at the edge of the cell is several seconds. The high dynamics of the actin network provides it the ability to play multiple roles in cell mobility, maintaining cell shape, signal transduction, cell adhesion, transcription and muscle contraction, and the formation of a contractile ring during cytokinesis [1,2].

Six actin isoforms are present in the cells of vertebrates—four predominantly tissue-specific muscle isoforms (skeletal, cardiac, and smooth muscles) and two non-muscle isoforms (named cytoplasmic β-actins and γ-actins)—that are essential for almost all the cells [2,3]. The ratio and subcellular distribution of actin isoforms are variable and depend on the cell type [4,5,6].

Despite the long research history, it is still not clear why two such similar non-muscle actin isoforms coexist in the cell. The following questions remain open: (1) Do non-muscle actins have distinct functions in the cytoplasm of the same cell and at the different stages of the cell cycle? (2) Is the localization of non-muscle actins essential for the normal architecture of the cytoskeleton and organization of the intracellular space? (3) What is the contribution of non-muscle actins to the progression of the pathological processes, including the cell transformation? This is especially important for understanding the function of cytoplasmic actins in endothelial cells, since angiogenesis and endothelial-to-mesenchymal transition plays an essential role in pathogenesis of chronic pulmonary and vascular human diseases.

## 2. Cytoplasmic Actin Isoforms: Structures and Functions in the Interphase Non-Muscle Cells

The family of actin proteins is highly conserved. The greatest difference is observed in the amino acid sequence of muscle and non-muscle isoforms [2]. Two cytoplasmic actins and many actin-binding proteins, which provide the organization of various structures, form the microfilament cytoskeleton of non-muscle cells. The amino acid sequences of cytoplasmic β-actin and γ-actin (hereafter β-actin and γ-actin) differ only in four residues located at the N-terminus of the polypeptide chain [3].

**Figure 1:**
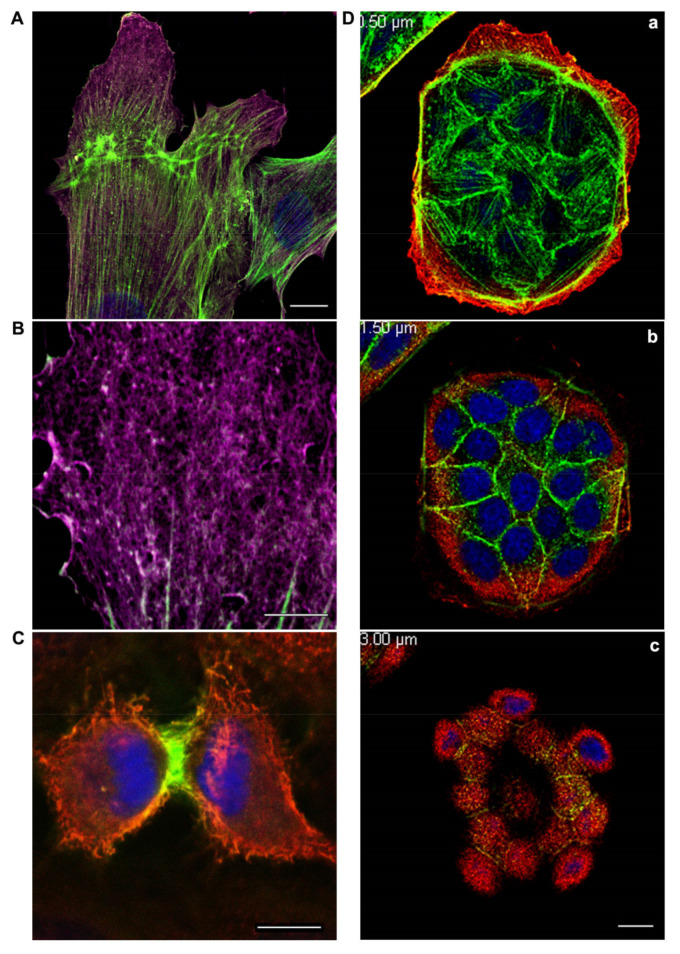
(**A**,**B**): β-actin and γ-actin display distinct distribution at the leading edge of fibroblasts moving towards the wound. An experimental wound was performed by scratching a monolayer of normal human subcutaneous fibroblasts. The cells were fixed and stained for cytoplasmic actins three hours later after scratching. β-actin bundles (green) and γ-actin network (purple) are pronounced in the motile cells. Laser scanning microscopy (LSM). Scale bars: (**A**) 10 μm; (**B**) 5 μm. (**C**): Segregation of cytoplasmic actin isoforms in cytokinesis of normal mitotic epithelial cells. β-actin and γ-actin are spatially segregated in cytokinesis of epithelial HaCaT cells. β-actin (green), γ-actin (red), and DNA (blue). LSM. Scale bar: 5 μm. (**D**): β-actin and γ-actin are localized in different structures and cell compartments. MDCK epithelial cells were cultivated for 3 days, fixed, and stained for cytoplasmic actins. β-actin (green) is present in basal bundles (**a**) and cell–cell contacts (**b**). γ-actin (red) is present in lamellar (**a**) and dorsal/cortical (**b**,**c**) networks. LSM. Scale bar: 10 μm.

β-Actin gene is essential for survival during embryonic development of mammals. Embryos of mice lacking β-actin are much smaller in size and die at the early stages of development [7]. Embryos without γ-actin pass the prenatal period of development with some delay and they experience an increased mortality rate after birth [8]. Mouse embryonic fibroblasts with β-actin knockout show reduced motility compared to normal cells [7]. There is a pronounced compensatory expression of α-smooth muscle actin and activation of the Rho signaling pathway in these fibroblasts. ROCK inhibitors restored motility of cells without β-actin. γ-Actin is an important structural element and positive regulator of cell migration. Knockdown of γ-actin results in excessive phosphorylation of cofilin and myosin light chain, which indicates ROCK activation, increased contractility, and inhibition of the cell motility [9,10,11,12]. Morphological studies of the structures that are formed by non-muscle actins became possible due to highly specific monoclonal antibodies against β-actin and γ-actin and the method of confocal microscopy [9]. In the non-muscle cells of different origin (epithelial, endothelial, and fibroblasts) β-actin and γ-actin organize different cytoskeletal structures that are diversely located within the cell, and can perform distinct functions [9,10,13]. In fibroblasts, β-actin is predominantly located in the stress fibers and in the focal contacts area; cortical and lamellar branched actin network consists of γ-actin (Figure 1A,B). In the lamellipodia, the colocalization of β- and γ-actin is observed (Figure 1B). β-Actin filaments are involved in the processes of cell contraction. In the epithelial cells, β-actin forms basal microfilament bundles and participates in the adhesion junctions; γ-actin organizes the cortical (dorsal) network of actin filaments (Figure 1D) and some stress fibers [9].

**Figure 2:**
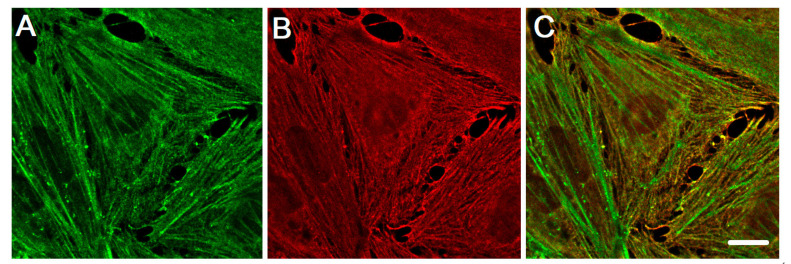
The distribution of cytoplasmic actin isoforms in the cytoplasm of endothelia cell in vitro. β-actin and γ-actin are spatially segregated in human pulmonary aorta endothelial cells. (**A**) β-actin (green), (**B**) γ-actin (red), and a merge of β-actin (green) and γ-actin (red) images (**C**). Structured Illumination Microscopy (SIM). Scale bars: 5 μm.

**Figure 3:**
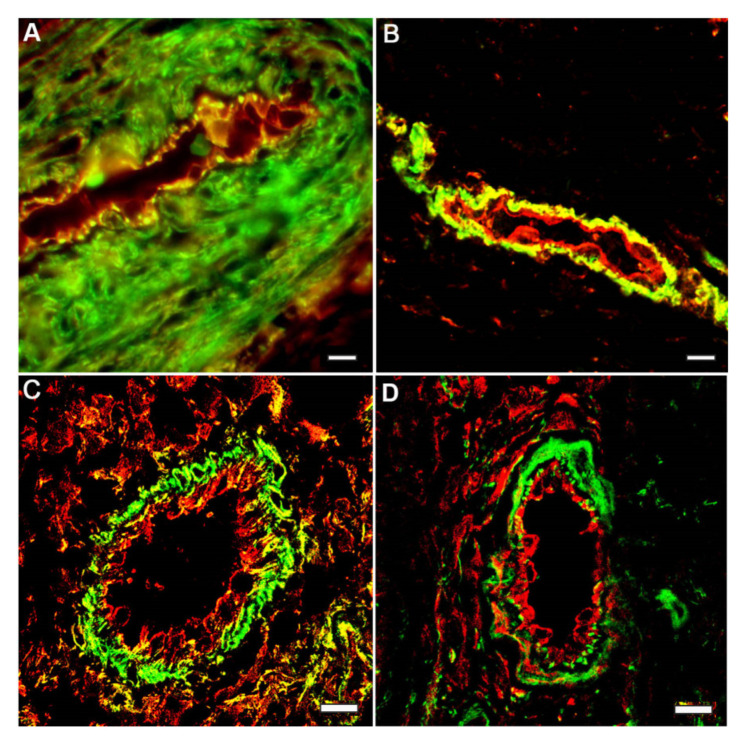
Segregation of cytoplasmic actin isoforms in the endothelia cell in vivo. Capillaries in tissue sections: longitudinal (**A**,**B**) and cross (**C**,**D**) tissue sections of the wound healing areas of stratified epithelium of human skin. Immunofluorescence staining of β-actin (green) and y-actin (red): **A**–**C**; β-actin (green) and vimentin (red): **D**. LSM. Scale bars: 5 μm.

The distribution of β-actin and γ-actin in the endothelial cells is similar to epithelial cells [14]. In endothelial cells, the actin cytoskeleton is represented by actin stress fibers, which include a cortical network of actin fibers and a membrane skeleton. Moreover, in the cells of other tissues, β-actins and γ-actins are segregated in the cytoplasm of endothelial cells in vitro (Figure 2) and in vivo (Figure 3). They form different types of intracellular structures in endotheliocytes. In endothelial cells of the human pulmonary artery (HPAEC) and cells of the human umbilical vein (EAhy926), in vitro β-actin mainly forms stress fibers and rounded microparticles in the cytoplasm of cells, while the cells consisted of γ-actin in the branched actin network in the cortical regions [15,16].

In the endothelial cells lining the vessel walls in a living organism, β-actin and γ-actin structures are also spatially separated. It was shown that endothelial cells of large vessels have very similar cytoskeleton organization, in contrast to microvascular endothelium [15,16,17]. The distribution (but not the density) of β-actin and γ-actin structures in the endotheliocytes of human artery and veins does not differ.

β-Actin is important for the structure and functional regulation of the adhesion junctions in epithelial cells and determines an apical-basal cell polarity. γ-Actin is associated with tight junctions in the epithelial cells. Suppression of β-actin causes the loss of intercellular contacts in the epithelial cells, while a downregulation of γ-actin induces an epithelial-myofibroblast transition (EMyT) that is accompanied by an increase in stress fibers and enhanced expression of α-smooth muscle actin [10,13].

Cytoplasmic β-actins and γ-actins are also involved in the endothelial barrier function [17]. β-Actin is found mainly in the stress fibers, while γ-actin forms a branched cortical network in the interphase endothelial cells of large vessels [15]. Coordinated rearrangements of β-actin and γ-actin filaments contribute significantly to the development of endothelial microparticles [14]. Endothelial microparticles are membrane vesicular structures released upon endothelial cell activation or the induction of apoptosis [18,19]. Both actin isoforms are responsible for the endothelial barrier function and the dynamics of cell contacts. However, β-actin is vital for endothelial cells: β-actin knockout results in the death of almost all endothelial cells [20]. The connection of the actin structures with microtubules (MT) is important for the functional activity of endothelial cells. Microfilament–MT interaction provides compression and relaxation of the cell during the endothelial barrier function. Thrombin or nocodazole treatment impairs the barrier function, induces MT disassembly, and results in the formation of large stress fibers [21]. MT affects the actin filament organization via local changes of actomyosin contractility at the end of stress fibers [22]. Actin filaments interact with the dynamic MT [21,23], which are the majority in endothelial cells [21]. The dynamics of MT is γ-actin-depended, which suggests the presence of a mechanical joint between γ-actin and MT [24]. This joint is possibly established through the intermediates, which may be isoform-specific. For example, MT plus-end binding protein EB1 is known to interact mainly with γ-actin, but not β-actin [25].

## 3. Different Impact of Actin Isoforms on the Process of Cell Division

Cytoplasmic actins are segregated in anaphase-telophase of normal mitotic epithelial cells [9,26]. The organization and functions of β-actins and γ-actins at different phases of mitosis of non-tumor epithelial cells were studied using laser scanning microscopy (LSM) [26]. It was shown that β-actins and γ-actins are spatially separated in early prophase, anaphase, telophase, and during cytokinesis (Figure 1C). A decrease in β- or γ-actin expression via small interference RNAs (siRNAs) results in a significant reduction in cell population. A decrease in β-actin causes the generation of multinucleated cells, which indicates a possible cytokinesis failure in these cells. Suppression of γ-actin expression diminishes the number of mitoses. The interdependence between actin isoforms and the MT system during mitosis is observed: The reduction in γ-actin induces the disorganization of the mitotic spindle, and suppression of tubulin polymerization influences β-actin arrangement. The role of actin isoforms is fundamentally different in the process of daughter cells separation: The contemporaneous production of a β-actin contractile ring at the cell equator and the loss of γ-actin from the poles are required to generate a stable cytokinetic furrow and for the completion of cell division [27]. Thus, both actin isoforms are required for normal cell division, but each isoform has its specific contribution to this process.

Actin cytoskeleton is reorganized during tumor transformation. This reorganization provides motility, invasion, and metastasis of tumor cells. Cytoplasmic actins play different roles in neoplastic transformation. The predominance of β-actin by exogenous expression induces epithelial differentiation and suppresses cell growth in culture, experimental invasion, and tumor xenografts growth of colon, lung, and mammary gland carcinoma cells. Thereby, β-actin acts as a tumor suppressor in the epithelial tumor cells of different tissue origin. On the contrary, γ-actin enhances malignant features of epithelial tumor cells [28,29]. The depletion of each cytoplasmic actin results in impaired proliferation/cell cycle in carcinoma cells [28,29].

The cytoplasmic actins play distinct roles in cell cycle regulation of breast cancer cells. Downregulation of each cytoplasmic actin isoform inhibits the proliferation of breast cancer cells, but only suppression of β-actin stimulates the expression of cyclins A2, B1, and D3, whereas suppression of γ-actin reduces expression of these cyclins. γ-Actin is co-localized with extracellular signal-regulated kinases 1/2 (ERK1/2) in breast cancer MCF7 cells. The reduction in β-actin induces ERK1/2 activation, while γ-actin downregulation inhibits phosphorylation of ERK1/2. ERK1/2, γ-actin, and cyclin A2 directly interact in the same protein complex. The reduction in γ-actin results in a decrease in cyclin A2, inhibits ERK1/2 signaling, and inhibits cell proliferation [28]. Nevertheless, quantitative differences in severity are possible even in carcinomas of the same tissue origin. Perhaps this is due to the initial ratio of isoforms and different levels of ERK activation, which results in different sensitivity toward RNA interference or the effect of low molecular weight inhibitors of signaling pathways.

## 4. Crucial Involvement of Cytoplasmic Actins in the Endothelial Cell Motility and Angiogenesis

Both non-muscle actin isoforms are involved in the implementation of the barrier function, but they also play distinct roles in the other functions of endothelial cells. The migration of endothelial cells is essential for the blood vessels formation and Rho family GTPases participate in this process. The activation of Rac1 GTPase mediates the migration of endothelial cells by the actin cytoskeleton reorganization [30]. Cytoplasmic γ-actin plays a key role in endothelial cell motility and chemotaxis. The addition of fetal calf serum (FCS), fibroblast growth factor β (FGFβ), or endothelial cell growth factor (ECGF) significantly increases the endothelial cell motility [20]. This effect is not observed in the cells transfected with siRNA to γ-actin. Knocking down γ-actin expression had no significant effect on adhesion but strongly decreased endothelial cell motility and migration abilities. This effect was associated with an accumulation of thick actin stress fibers, large focal adhesions, and increased phosphorylation of myosin regulatory light chain, which suggests activation of the ROCK signaling pathway [20]. Remarkably, γ-actin knockdown cells were able to initiate morphological differentiation into capillary-like tubes but these structures were not formed completely and rapidly regressed. Incubation with H-1152 and Y-27632 and ROCK inhibitors, completely rescued the γ-actin knockdown-induced motility phenotype but not the angiogenic potential of endothelial cells [20]. These observations suggest that γ-actin plays a crucial role in angiogenesis through both ROCK-dependent and ROCK-independent mechanisms [20].

The participation of γ-actin in the both Rho-dependent and Rho-independent angiogenesis was demonstrated by Pasquier and colleges [20] and the fact that the knockdown of γ-actin gene does not affect the ability of endothelial cells to attach to various substrates (fibronectin, laminin, and collagen-1, for example) means that γ-actin is not involved in interaction with focal contacts.

## 5. Endothelial-to-Mesenchymal Transition in Cardiovascular Diseases: More Questions Than Answers

Interaction of actin isoforms with other components of the cell contractile apparatus is crucial for the cell transformation and tumorigenesis process. The contribution of β-actins and γ-actins to the processes of tumor transformation is well investigated [28]. A decrease in γ-actin via siRNA induces a contractile phenotype in non-cancer epithelial cells [9] and results in the normalization of the phenotype of carcinoma cells [28]. Loss of cytoplasmic γ-actin stimulates formin-mediated actin polymerization and the activation of Rho GTPase, which apparently causes the epithelial-to-myofibroblast transition and is accompanied by a decrease in cell motility [10]. When it comes to endothelial cells, there is an extremely interesting and still unclear transformation process of endothelial-to-mesenchymal transition, which results in many pathologies.

Endothelial-to-mesenchymal transition (EndoMT) is a cell transdifferentiation process where endothelial cells progressively lose endothelial specific markers (e.g., VE-cadherin and CD31) and obtain mesenchymal markers (e.g., vimentin, fibronectin, and α-SMA) and mesenchymal phenotype. This phenomenon was first discovered in the embryonic development of the heart [31]. Accumulating evidence shows that EndoMT is implicated in the pathogenesis of chronic lung diseases, including pulmonary hypertension and lung fibrosis [32,33,34].

However, the mechanisms underlying EndoMT and its role in these lung diseases are not fully understood. It is known that the breakdown of the complete epithelial junctional complex initiates the loosening of cell–cell contacts and promotes the transition of epithelial cells to mesenchymal cells [35].

It was shown that the majority of signaling networks that are commonly involved during epithelial-mesenchymal transition are also responsible for EndoMT [36]. The principle mediators are the transforming growth factor-β (TGF-β) superfamily of proteins, including isoforms TGF-β1, TGF-β2, as well as bone morphogenetic proteins (BMPs BMP2, BMP4, BMP6, BMP9, and BMP10) [37,38,39,40]. Wingless/Integrated(Wnt)/β-catenin and Notch signaling pathways also activate EndoMT [41,42]. These signaling pathways interact with transcription factors (Snail, Slug, Zinc Finger E-Box Binding Homeobox 1 (ZEB1), ZEB2, and TWIST1), which induce mesenchymal cell gene expression but suppress endothelial gene expression in endothelial cells [36]. Furthermore, BMP/TGF-β, Wnt/β-catenin and Notch pathways can cross-regulate each other during the EndoMT [43,44]. TGFβ (TGFβ/Smad3/Snail axis) involvement into EndoMT development was shown on the rat model (as in vivo animal model) and in the primarily cultured rat pulmonary microvascular endothelial cells (as in vitro cell model) [45]. TGF-β induces EndoMT in Fibrotic Diseases [46].

Disturbed shear stress and matrix stiffness are of great importance to EndoMT [40,47,48,49]. Shear stress regulates endothelial cell function and phenotype via mechanochemical signal transduction [48,49,50]. Mechanistically, laminar shear stress activates MEK5/ERK5 cascade and fibroblast growth factor (FGF) receptor 1 thereby repressing EndoMT. Vascular stiffness results from excessive matrix deposition within the vessel wall, which commonly accompanies pulmonary and vascular diseases such as fibrosis, hypertension, and atherosclerosis. Vascular stiffness triggers EndoMT [47,51], the mechanisms are associated with β-catenin activation.

With pulmonary hypertension, the number of α-SMA positive mesenchymal-like cells are increased in obstructive pulmonary vascular lesions [32]. Recent studies have demonstrated that EndoMT is an important contributor of α-SMA positive cells in patients with pulmonary hypertension [32,33,34]. Several pathways including TGF-β and Wnt/β-catenin are also involved in pulmonary hypertension associated EndoMT [32,33,52].

TGF-β induces EndoMT in Fibrotic Diseases [46]. Microvascular endothelial cells (HMEC-1) undergoing EndoMT adopt an intermediate state of drifting migration model between the mesenchymal and amoeboid protrusive types in the early stages of fibrosis. HMEC-1 cells gained an intermediate mesenchymal phenotype in response to TGF-β2 treatment. Migration of HMEC-1 undergoing EndoMT is dependent on Rho/ROCK cytoskeleton contraction [53].

Finally, it was shown that EndoMT inhibitors directly upregulate the endothelial-specific adhesion molecule VE-cadherin [54]. VE-cadherin adherent junctions directly interact with actin filaments and MT which suggests the involvement of these cytoskeletal structures into EndoMT.

The analysis of available data suggests the involvement of endothelial permeability regulators that affect cytoskeletal and adhesive structures and barrier function, including the universal regulators of the cytoskeleton-Rho/ROCK family GTPases in EndoMT. However, the involvement of individual components of the cytoskeleton (in particular, actin structures) during EndoMT is still unclear. This is an extremely promising field since EndoMT, MT, and actin structures share the regulating molecular cascades.

Drawing an analogy with the epithelial-to-myofibroblast transition, it can be assumed that the participation of actin isoforms is probably essential for the EndoMT process. Investigation of the cytoplasmic actins influence on EndoMT can become a key factor in the understanding of this pathology development as well as cardiovascular and fibrotic diseases.

## 6. Conclusions and Perspectives

The functions of non-muscle β- and γ-actins have been investigated for a long time, using the molecular and cell biology methods, in particular silencing or overexpression of genes encoding these isoforms [8,9,55,56,57,58,59]. The data have been frequently contradictory, which could be associated with technical difficulties of experiments, including a different level of selective suppression or overexpression of each actin isoform.

Today we consider that β-actins and γ-actins form distinct structures in the cytoplasm of non-muscle cells of various origin. The distributions within the cell and functions of isoform-specific actin structures are distinguishable at the different stages of the cell cycle in interphase and during mitosis [10,17,26]. The differences in the polymerization of non-muscle actins [2,60] and isoform-specific interaction with proteins that regulate polymerization [25,28,61,62] enables the supposition that cytoplasmic actins perform distinct functions in the cell [9,10,12,13,17,25,29]. The contribution of actin isoforms to the development of pathological processes, including tumor transformation, is different [10,11,12,28,29,63].

Both cytoplasmic actin isoforms are fundamentally important for the vital activity of the cells and each isoform has specific functions despite the minimal structural differences. The ratio and interaction of actin isoforms are necessary for the normal functioning of interphase and mitotic cells and imbalance results in the development of pathologies, including tumor transformation and transition.

Actin isoforms have tissue-specific functional features that depend on the cell type. Endotheliocytes cytoplasmic β-actins and γ-actins are involved in the endothelial barrier function and both actin isoforms are responsible for the dynamics of cell contacts; however, β-actin is vital for endothelial cells. The connection of the actin structures with MT is significant for the functional activity of endothelial cells. Microfilament–MT interaction provides compression and relaxation of the cell during the endothelial barrier function and cell–substrate contacts; VE-cadherin adherent junctions are also involved. By focusing on the role of actins in the functional activity of endothelial cells, the contribution of cytoplasmic actins to angiogenesis and EndoMT are promising for further investigations, for fundamental cell biology, and for practice medicine.

## Data Availability

The data presented in this study are available in https://pubmed.ncbi.nlm.nih.gov/.
